# Supplementary material for: Circulation of Salmonella spp. between humans, animals and the environment in animal-owning households in Malawi
Source: Nat Commun. 2025 Nov 12;16:9703. doi: 10.1038/s41467-025-65266-1 (PMC12612273; doi:10.1038/s41467-025-65266-1)
Supplement: Supplementary file 1 — Supplementary Information [file 41467_2025_65266_MOESM1_ESM.pdf]

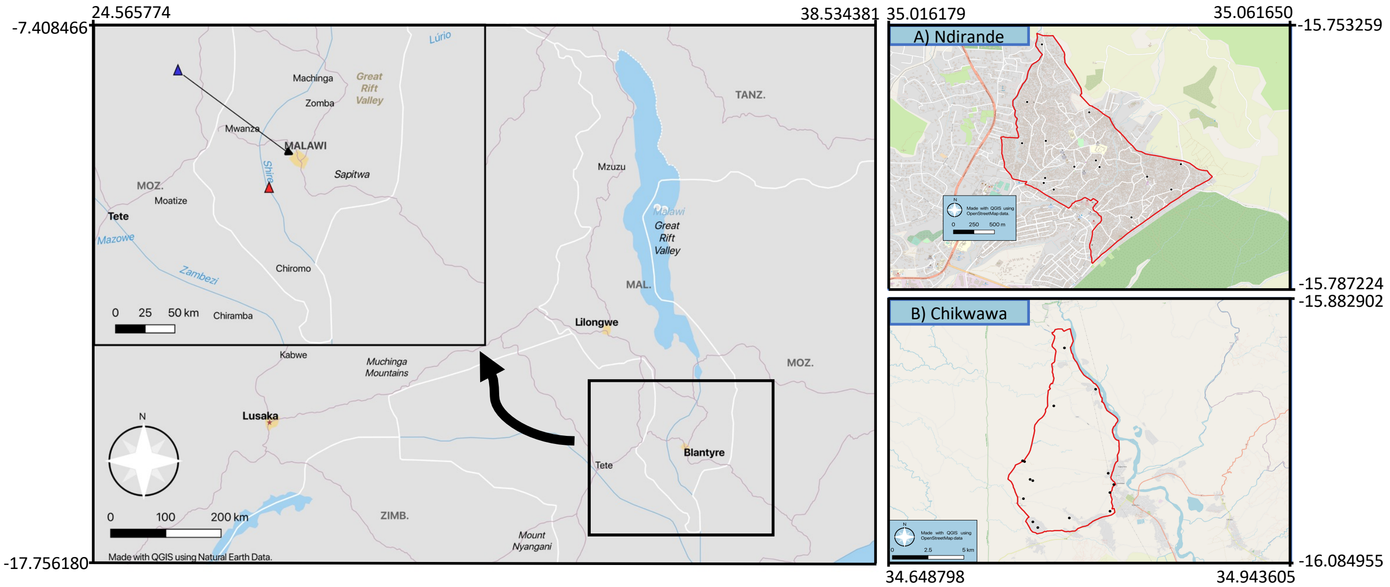

Supplementary Figure 1; Location of households sampled in each of the study sites within Malawi and polygons of A) Ndirande, B) Chikwawa study sites. *Red line=edge of study site sampling polygon. Black dot=location of household enrolled in study. Red triangle = location of Chikwawa study site. Blue triangle = location of Ndirande study site. Black rectangles and arrow depict a broader and closer view of the study sites. Latitude and longitude detailed of outside edges of plots. ©OpenStreetMap® contributors* (https://www.openstreetmap.org/copyright) OpenStreetMap® is *open data*, licensed under the Open Data Commons Open Database License (ODbL) by the OpenStreetMap Foundation (OSMF) and the Creative Commons Attribution-ShareAlike 2.0 license (CC BY-SA 2.0).

Supplementary Figure 2; Schematic outline of study laboratory protocol for Salmonella *isolation*

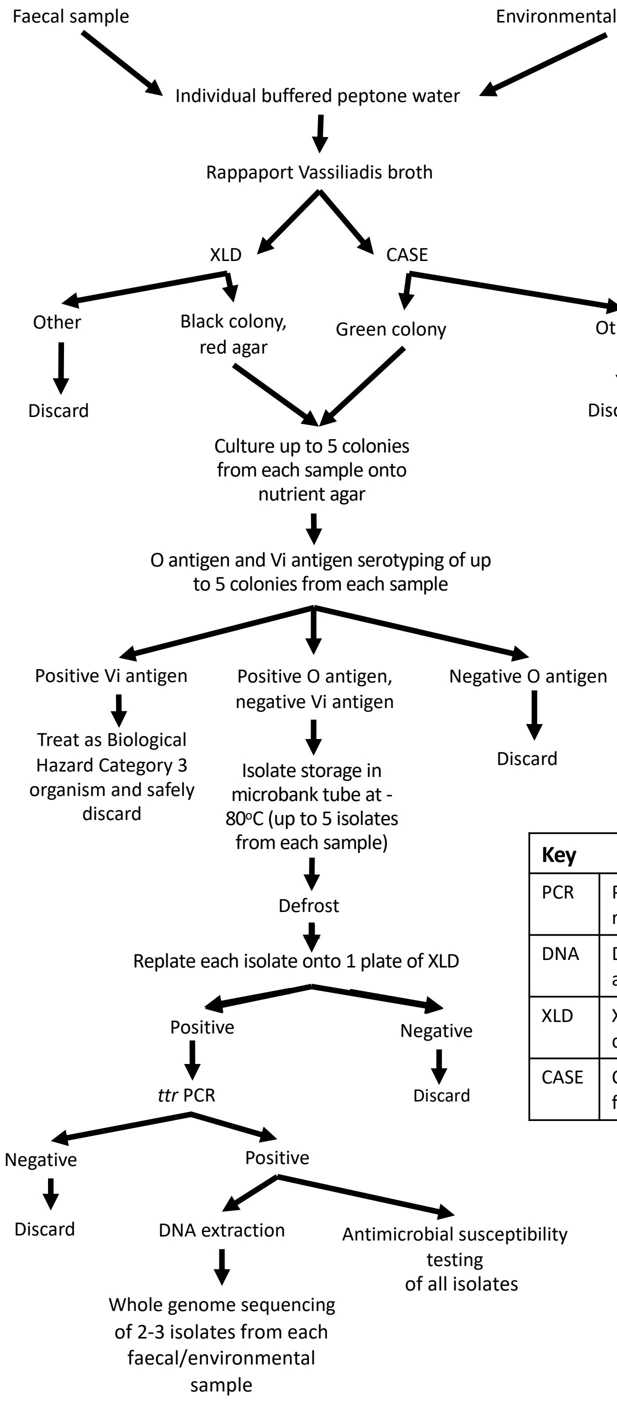

**Key**	
PCR	Polymerase chain reaction
DNA	De-oxyribonucleic acid
XLD	Xylose lysine deoxycholate agar
CASE	Chromogenic agar for *S.* Esterase

Supplementary Figure 3; The distribution of* S. enterica *and* S. salamae *genomes by serovar predicted by SISTR using Pathogenwatch. Source data are provided as a Source Data file.

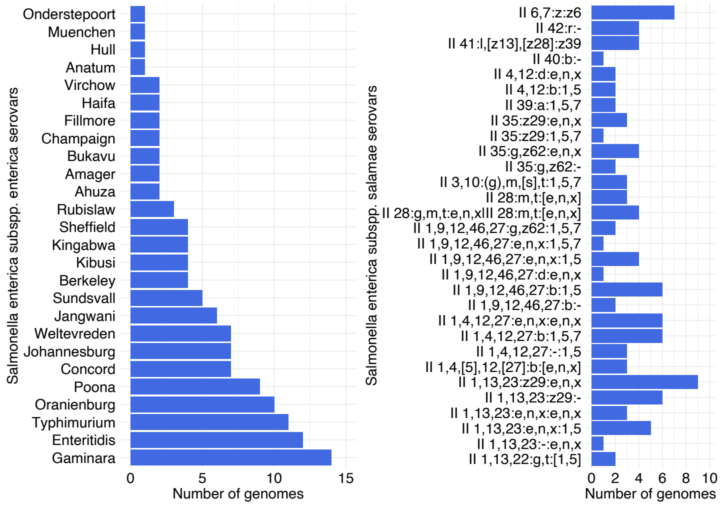

Suppl Figure 4; AMR genes and plasmid replicons mapped to the phylogeny. *Black bars = chromosomal quinolone resistance, red bars = other AMR genes, green = plasmid replicons. Maximum likelihood phylogenetic tree built using RAxML and visualised with ggtree. rc = rep cluster, Subspecies: yellow =* S. enterica*, purple =* S. salamae. *Source data are provided as a Source Data file.*

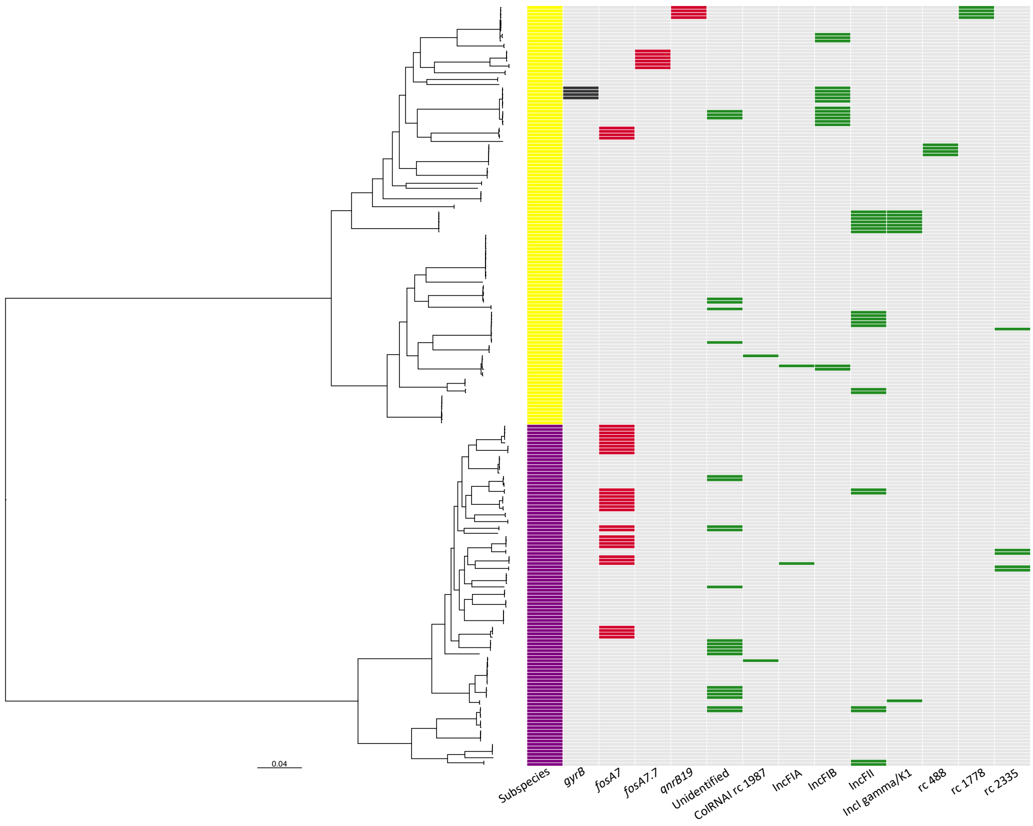

Suppl. Figure 5) With duplicates

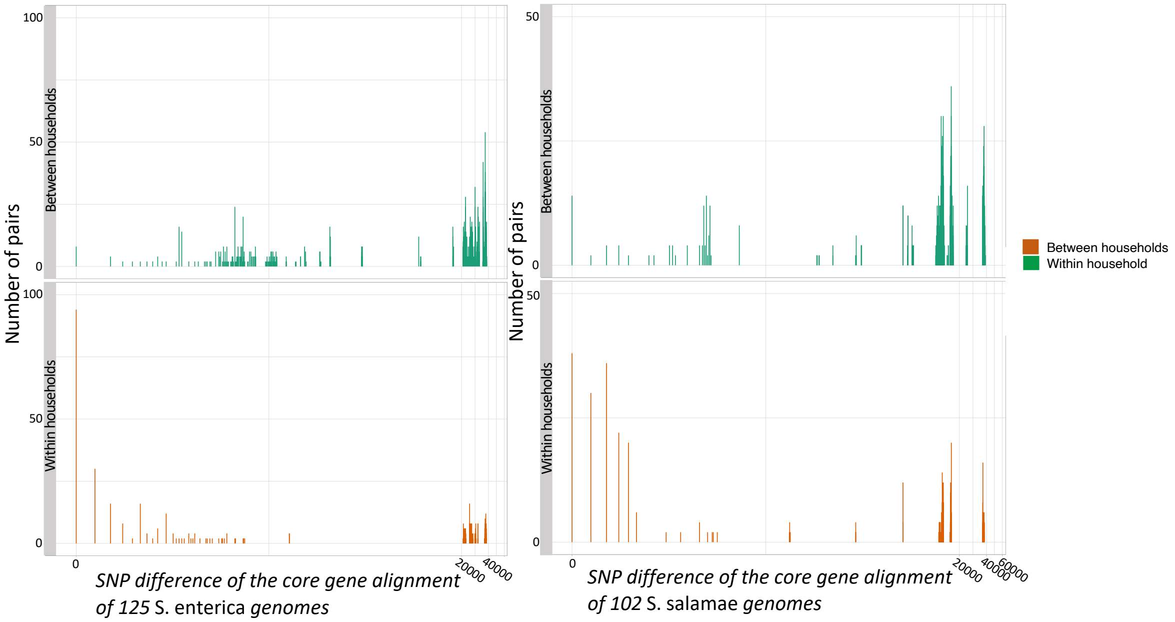

Supplementary Figure 5 and 6; Pairwise SNP distance of collection of 125 S. enterica genomes and 102 S. salamae genomes. *Figure 5 shows entire collection, including within host diversity. Figure 6 depicts collection once within host diversity removed. Source data are included as a Source Data file.*

Suppl. Figure 6) Duplicates removed.

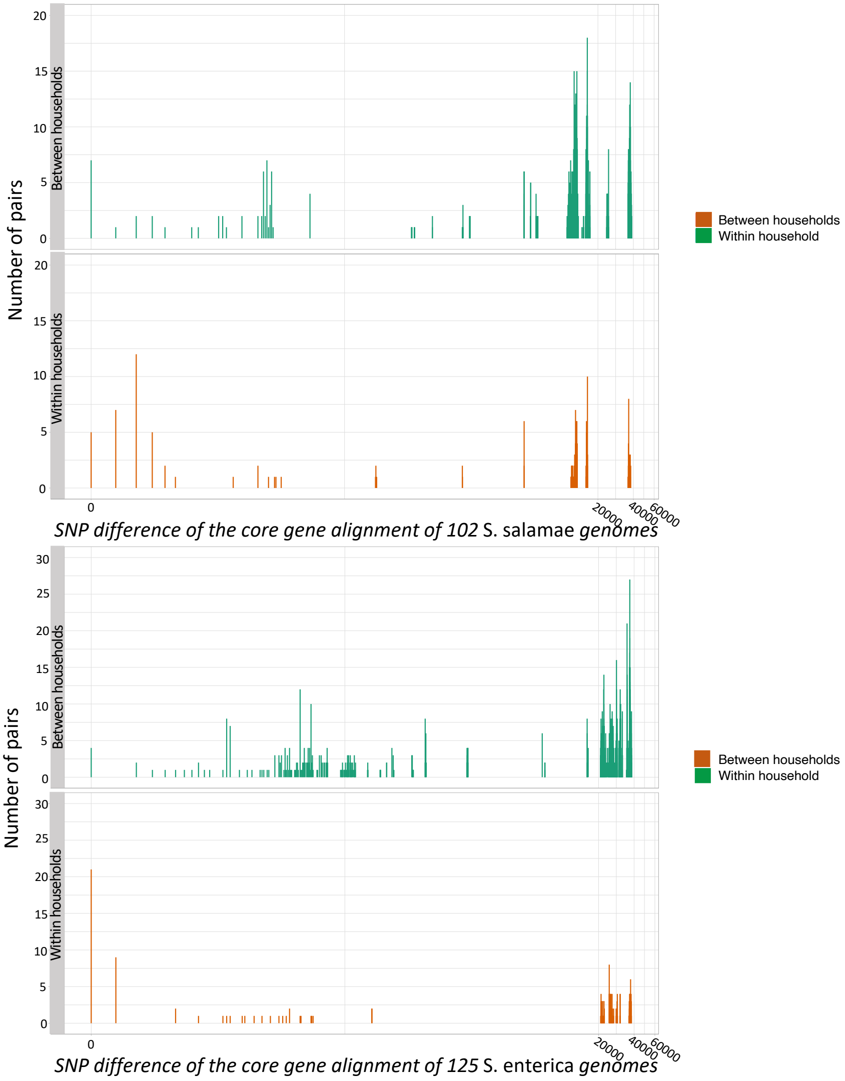

Supplementary Figure 7; Study isolates of* S. *Typhimurium ST313 included in a maximum likelihood RAxML phylogenetic tree containing currently recognised lineages of ST313 constructed using a mapping alignment. *Red arrow = reference genome* S. *Typhimurium ST313 D23580. Rooted to* S. *Typhimurium ST19 LT2. Invasive = isolated from blood, cerebrospinal fluid or bone. Source data are included as a Source Data file.*

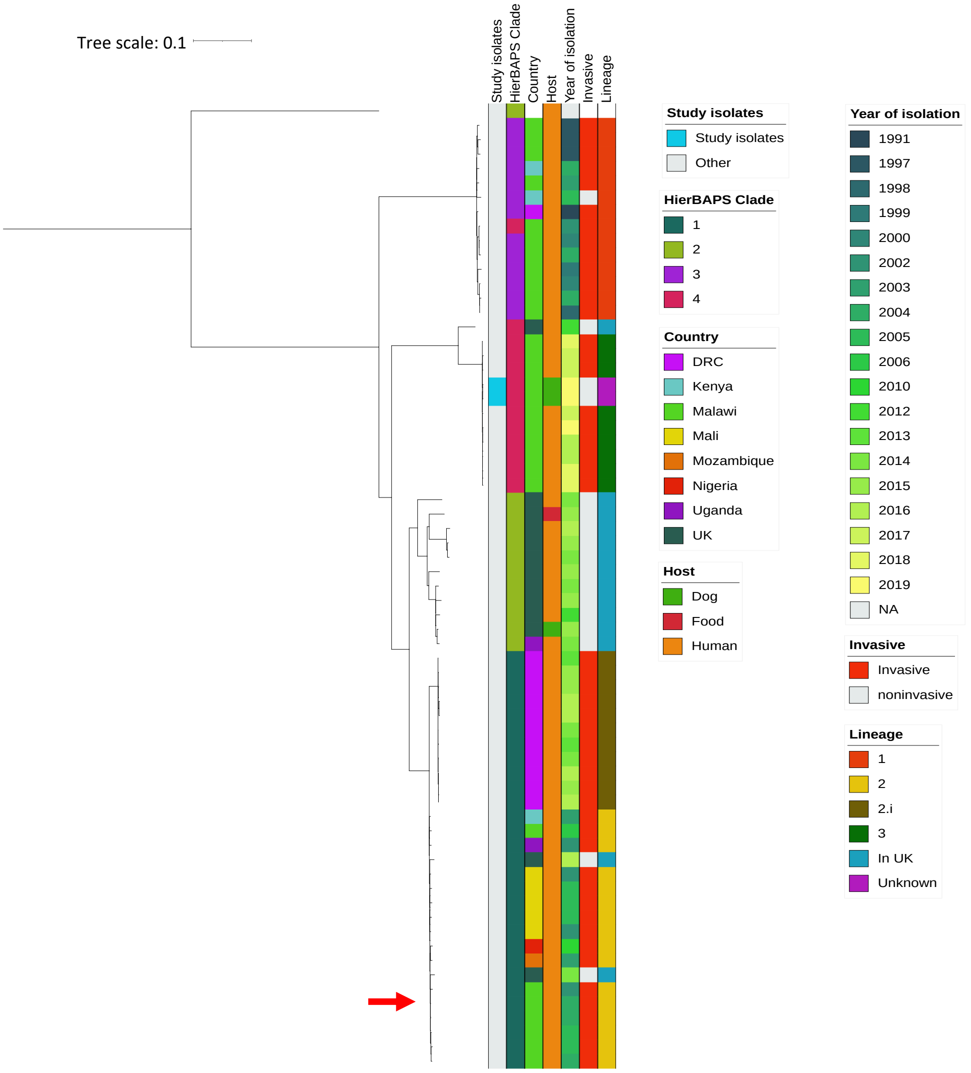

Supplementary Figure 8; Maximum likelihood RAxML phylogenetic tree constructed using a mapping alignment using ITOL to place the S. Enteritidis ST11 isolated within this study in the context of previously sequenced sub-Saharan African isolates of the same sequence type. *This tree is rooted to* S. *Gallinarum. S. Enteritidis P125109 is used as a reference genome, shown by the red arrow. HierBAPS clade column show rhierBAPS clades as assigned within this study. Epidemic clades as described by Feasey are used to colour the tree. Invasive = isolated from blood, cerebrospinal fluid or bone. Source data are included as a Source Data file.*

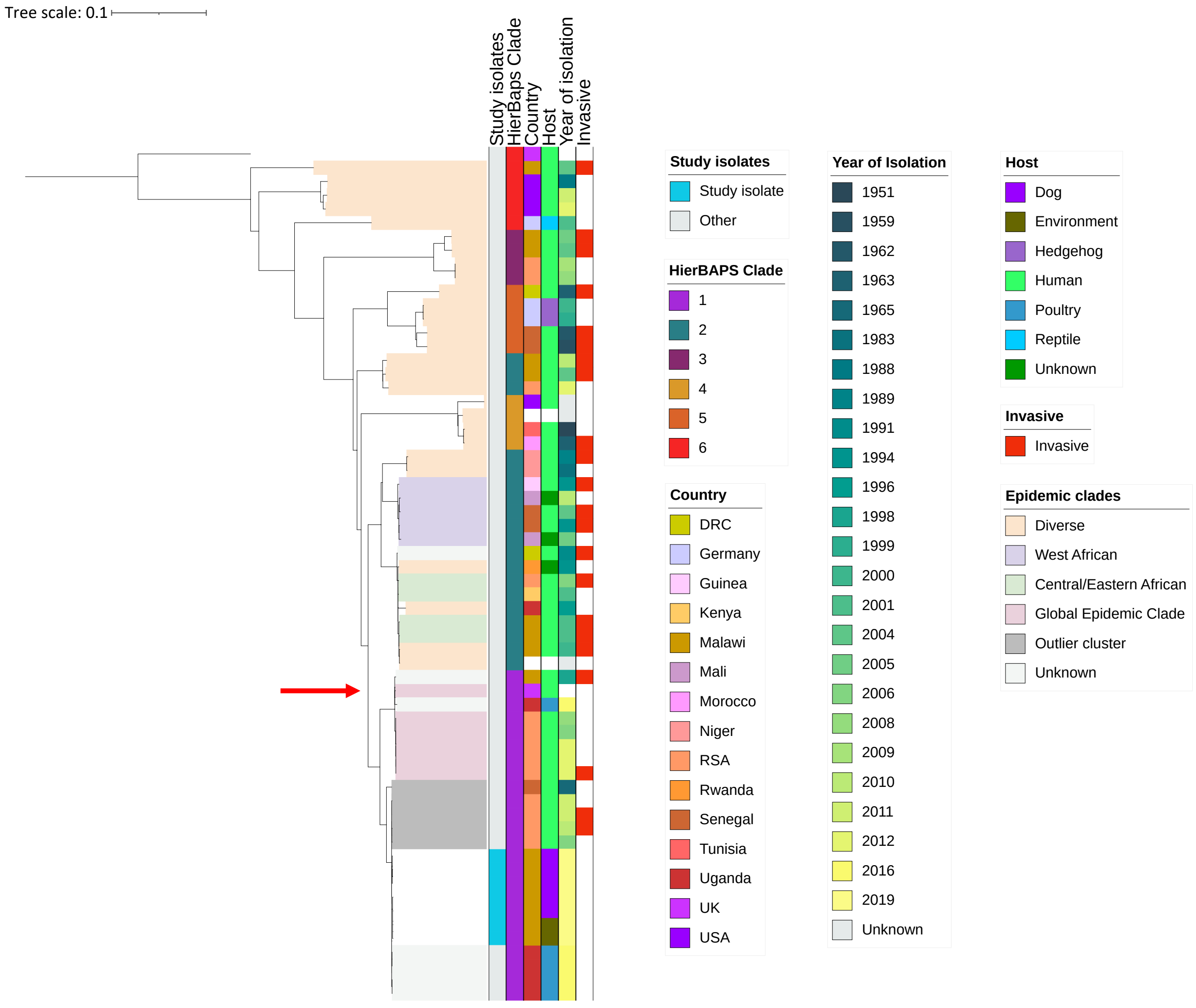

Supplementary Table 1; Reaction mix used for PCR reaction

	**µl/reaction**	**µl/94 reactions**
Sample	2.5	235
Takyon Master Mix Low ROX	12.5	1,175
ttr forward and reverse primer	1	47 each primer
ttr probe	1	94
Nuclease free water	8.0	752
Total	25	2350

Supplementary Table 2; DNA sequence of primers used

**Designation**	**Sequence**	**Melting Temperature (°C)**	**Modification**
ttr-6 (forwards)	CTCACCAGGAGATTACAACATGG	57	
ttr-4 (backwards)	AGCTCAGACCAAAAGTGACCATC	58	
ttr P	CACCGACGGCGAGACCGACTTT		5’: 6FAM & 3’: BHQ-1

Supplementary Table 3; Total number of samples taken each study site demonstrating the total number which were PCR positive.

	**Ndirande**	**Chikwawa**	**Overall**
Total number of samples taken	965	1,115	2,080
Total individual samples PCR positive (n(%))	87(9.0%)	146(13.1%)	233(11.2%)
Total picks of *Salmonella* PCR positive	237	631	838
Average number of picks PCR positive stored from a single sample (n(range))	2.7(1-8)	4.3(1-10)	3.6

Supplementary Table 4; Parameters used as cut-offs for quality control procedures. (Mbp = mega basepairs).

**Quality control programme**	**Parameter**	**Value for exclusion**
Kraken	Proportion reads	<70% abundance *Salmonella*
CheckM	Contamination	>20% contamination<br><90% completeness
Assembly stats	Genome length<br>Contigs	>7,000,000bp<br>>500
Quast	Contigs<br>N50<br>Total bp	>500<br><20kb<br><4Mbp, >5.8Mbp
Pathogenwatch	Serovar	Remove bacterial species not *Salmonella*
