# Supplementary material for: Circulation of Salmonella spp. between humans, animals and the environment in animal-owning households in Malawi
Source: Nat Commun. 2025 Nov 12;16:9703. doi: 10.1038/s41467-025-65266-1 (PMC12612273; doi:10.1038/s41467-025-65266-1)
Supplement: Supplementary file 2 — Reporting Summary [file 41467_2025_65266_MOESM2_ESM.pdf]

nature portfolio

Corresponding author(s): Catherine Nicole Wilson

Last updated by author(s): Sept 12, 2025

Reporting Summary

Nature Portfolio wishes to improve the reproducibility of the work that we publish. This form provides structure for consistency and transparency in reporting. For further information on Nature Portfolio policies, see our Editorial Policies and the Editorial Policy Checklist.

Please do not complete any field with "not applicable" or n/a. Refer to the help text for what text to use if an item is not relevant to your study.
For final submission: please carefully check your responses for accuracy; you will not be able to make changes later.

Statistics

For all statistical analyses, confirm that the following items are present in the figure legend, table legend, main text, or Methods section.

n/a	Confirmed	
☐	☒	The exact sample size (*n*) for each experimental group/condition, given as a discrete number and unit of measurement
☐	☒	A statement on whether measurements were taken from distinct samples or whether the same sample was measured repeatedly
☐	☒	The statistical test(s) used AND whether they are one- or two-sided<br>*Only common tests should be described solely by name; describe more complex techniques in the Methods section.*
☐	☒	A description of all covariates tested
☐	☒	A description of any assumptions or corrections, such as tests of normality and adjustment for multiple comparisons
☐	☒	A full description of the statistical parameters including central tendency (e.g. means) or other basic estimates (e.g. regression coefficient) AND variation (e.g. standard deviation) or associated estimates of uncertainty (e.g. confidence intervals)
☐	☒	For null hypothesis testing, the test statistic (e.g. *F*, *t*, *r*) with confidence intervals, effect sizes, degrees of freedom and *P* value noted<br>*Give P values as exact values whenever suitable.*
☒	☐	For Bayesian analysis, information on the choice of priors and Markov chain Monte Carlo settings
☒	☐	For hierarchical and complex designs, identification of the appropriate level for tests and full reporting of outcomes
☒	☐	Estimates of effect sizes (e.g. Cohen's *d*, Pearson's *r*), indicating how they were calculated

Our web collection on statistics for biologists contains articles on many of the points above.

Software and code

Policy information about availability of computer code

Data collection	Questionnaires detailing household composition, socioeconomic data, animal ownership, husbandry, contact of members with animals, health seeking behaviour of humans were administered at each household using an electronic case report form on a Samsung tablet device using Open Data Kit Collect version 1.18.
Data analysis	Custom code used for data analysis within this study is available at the following GitHub page: https://github.com/CatherineWilson511/Circulation-of-Salmonella/edit/main/README.md

For manuscripts utilizing custom algorithms or software that are central to the research but not yet described in published literature, software must be made available to editors and reviewers. We strongly encourage code deposition in a community repository (e.g. GitHub). See the Nature Portfolio guidelines for submitting code & software for further information.

Data

Policy information about availability of data

All manuscripts must include a data availability statement. This statement should provide the following information, where applicable:

- Accession codes, unique identifiers, or web links for publicly available datasets
- A description of any restrictions on data availability
- For clinical datasets or third party data, please ensure that the statement adheres to our policy

Raw sequencing reads for all novel sequences are deposited at the European Nucleotide Archive (ENA) under project PRJEB32657. All accession numbers (both

novel and previously published) used in this project are listed in the Supplementary Data along with all metadata used for analysis in Figure 1, 2, 3 and Supplementary Figures 3, 4, 5 and 6. Previously published contextual metadata used in Supplementary Figure 7 and Supplementary Figure 8 are displayed in the file 'Source Data- Household Circulation of Salmonella spp. between humans, animals and the environment in Malawi - .xlsx' Sheets ST313 Metadata and Mapping ST11. Publically available sequence data was downloaded from one of the following sources: GenBank (https:// www.ncbi.nlm.nih.gov/genbank/), Sequence Read Archive (https://www.ncbi.nlm.nih.gov/sra), European Nucleotide Archive (https://www.ebi.ac.uk/ena) or Enterobase (https://enterobase.warwick.ac.uk).

Human research participants

Policy information about studies involving human research participants and Sex and Gender in Research.

Reporting on sex and gender	Within this study in total 411 stool samples were collected from 184 humans. Human data was anonymised and information about sex or age was not analysed as part of this study as this information was not relevant to answer the proposed research question.
Population characteristics	Within this study in total 411 stool samples were collected from 184 humans. Human data was anonymised, and information about sex or age was not analysed or reported as part of this study.
Recruitment	Within each geographic area polygons were created using QGIS software to create areas for inclusion. Fifteen households were selected at random in the two study areas using R software version 2022.12.0+353 to generate random GPS coordinates using a spatial inhibitory design with close pairs. Households in each location met the inclusion criteria of being located within the study sites, all human household members were able to give informed consent or assent to take part in the study themselves, and the Head of the Household was able to provide informed consent to sample animals and the environment within the household. Households were excluded if a household member or representative was unable to provide informed consent, household members spoke neither Chichewa or English and if the household was located outside the boundary of the study sites.
Ethics oversight	Ethical approval for this study was obtained from the University of Liverpool Veterinary Research Ethics Committee (Reference number VREC686) and the College of Medicine Research Ethics Committee (COMREC), Malawi (Reference Number P.02/18/2368).

Note that full information on the approval of the study protocol must also be provided in the manuscript.

Field-specific reporting

Please select the one below that is the best fit for your research. If you are not sure, read the appropriate sections before making your selection.

☒ Life sciences ☐ Behavioural & social sciences ☐ Ecological, evolutionary & environmental sciences

For a reference copy of the document with all sections, see nature.com/documents/nr-reporting-summary-flat.pdf

Life sciences study design

All studies must disclose on these points even when the disclosure is negative.

Sample size	A sample size calculation was not performed due to the nature of the study. Households were chosen at random for inclusion into the study, and each household had a different composition of human and animal species, according to both the nature of the species and the number of each species present. Each different animal species has a different reported predicted prevalence of carriage of *Salmonella* within faeces and therefore calculation of a sample size for each household was impossible. According to this premise, the total number of households and the number of samples collected from each household was selected pragmatically and according to budget and time allowance to provide a picture of circulation of *Salmonella* within households within the study sites.
Data exclusions	Poor quality whole genome sequence data was excluded from the study. The cut-offs for exclusion of poor quality whole genome sequence data were pre-established according to current published recommendations.
Replication	A single microbiological culture of each sample collected was performed.
Randomization	Randomisation was not part of the study design of this longitudinal prospective cohort study.
Blinding	Blinding was not part of the study design of this longitudinal prospective cohort study.

Reporting for specific materials, systems and methods

We require information from authors about some types of materials, experimental systems and methods used in many studies. Here, indicate whether each material, system or method listed is relevant to your study. If you are not sure if a list item applies to your research, read the appropriate section before selecting a response.

Materials & experimental systems

n/a	Involved in the study
☒	☐ Antibodies
☒	☐ Eukaryotic cell lines
☒	☐ Palaeontology and archaeology
☐	☒ Animals and other organisms
☒	☐ Clinical data
☒	☐ Dual use research of concern

Methods

n/a	Involved in the study
☒	☐ ChIP-seq
☒	☐ Flow cytometry
☒	☐ MRI-based neuroimaging

Animals and other research organisms

Policy information about studies involving animals; ARRIVE guidelines recommended for reporting animal research, and Sex and Gender in Research

Laboratory animals	The study did not involve laboratory animal participants.
Wild animals	The study involved faecal sample collection from wild rodents, geckos and wild birds. Faeces were collected from samples of stool from these species which had been deposited around the households and therefore direct contact with wild animals was not necessary.
Reporting on sex	Sex of the animals from which faecal samples were collected was not recorded.
Field-collected samples	The study involved work with faecal samples collected from domestic and wild animals. These faecal samples, once collected, were transported back to the laboratories for microbiological culture. Samples were stored at 4°C with ice packs in a cool box until arrival at the College of Medicine (now KUHES) laboratories. Laboratory processing commenced within four hours of sample collection.
Ethics oversight	Ethical approval for this study was obtained from the University of Liverpool Veterinary Research Ethics Committee (Reference number VREC686) and the College of Medicine Research Ethics Committee (COMREC), Malawi (Reference Number P.02/18/2368).

Note that full information on the approval of the study protocol must also be provided in the manuscript.

This checklist template is licensed under a Creative Commons Attribution 4.0 International License, which permits use, sharing, adaptation, distribution and reproduction in any medium or format, as long as you give appropriate credit to the original author(s) and the source, provide a link to the Creative Commons license, and indicate if changes were made. The images or other third party material in this article are included in the article's Creative Commons license, unless indicated otherwise in a credit line to the material. If material is not included in the article's Creative Commons license and your intended use is not permitted by statutory regulation or exceeds the permitted use, you will need to obtain permission directly from the copyright holder. To view a copy of this license, visit http://creativecommons.org/licenses/by/4.0/
